# Herbivore Preference for Native vs. Exotic Plants: Generalist Herbivores from Multiple Continents Prefer Exotic Plants That Are Evolutionarily Naïve

**DOI:** 10.1371/journal.pone.0017227

**Published:** 2011-03-04

**Authors:** Wendy E. Morrison, Mark E. Hay

**Affiliations:** School of Biology, Georgia Institute of Technology, Atlanta, Georgia, United States of America; Northeastern University, United States of America

## Abstract

Enemy release and biotic resistance are competing, but not mutually exclusive, hypotheses addressing the success or failure of non-native plants entering a new region. Enemy release predicts that exotic plants become invasive by escaping their co-adapted herbivores and by being unrecognized or unpalatable to native herbivores that have not been selected to consume them. In contrast, biotic resistance predicts that native generalist herbivores will suppress exotic plants that will not have been selected to deter these herbivores. We tested these hypotheses using five generalist herbivores from North or South America and nine confamilial pairs of native and exotic aquatic plants. Four of five herbivores showed 2.4–17.3 fold preferences for exotic over native plants. Three species of South American apple snails (*Pomacea sp.*) preferred North American over South American macrophytes, while a North American crayfish *Procambarus spiculifer* preferred South American, Asian, and Australian macrophytes over North American relatives. Apple snails have their center of diversity in South America, but a single species (*Pomacea paludosa*) occurs in North America. This species, with a South American lineage but a North American distribution, did not differentiate between South American and North American plants. Its preferences correlated with preferences of its South American relatives rather than with preferences of the North American crayfish, consistent with evolutionary inertia due to its South American lineage. Tests of plant traits indicated that the crayfish responded primarily to plant structure, the apple snails primarily to plant chemistry, and that plant protein concentration played no detectable role. Generalist herbivores preferred non-native plants, suggesting that intact guilds of native, generalist herbivores may provide biotic resistance to plant invasions. Past invasions may have been facilitated by removal of native herbivores, introduction of non-native herbivores (which commonly prefer native plants), or both.

## Introduction

Exotic species disrupt native ecosystems and produce significant economic and environmental costs across all habitat types [1], [2], but impacts appear especially strong in freshwater ecosystems [3]. Numerous hypotheses focus on the processes underlying invasion success, the dynamics of establishment, and patterns of species spread [4], [5]. The enemy release hypothesis and the biotic resistance hypothesis are two prominent and divergent theories addressing how interactions between herbivores and plants may exacerbate or retard the establishment and spread of non-native plants.

The enemy release hypothesis postulates that non-native plants entering novel environments will escape their co-evolved, native enemies and that this escape frees resources and facilitates the spread of exotic plants [6], [7]. The biotic resistance hypothesis suggests that native species function as natural enemies (consumers, pathogens, competitors) of non-native invaders and suppress their establishment and spread in the new habitat [6], [8], [9]. Though commonly viewed as competing, these hypotheses need not be mutually exclusive [10]. When a non-native plant invades a new habitat, it will have escaped many of the specialist herbivores from its previous habitat (enemy release), but may also be encountering many new generalist herbivores that it will not have been selected to deter or tolerate (biotic resistance). The effects of herbivores on the invading plant may thus be determined by the net effect of escaping old herbivores and acquiring new ones. This net effect may depend on the relative impact of generalist versus specialist herbivores on plant fitness [10], the phylogenetic isolation of the plants (when native herbivores do not co-occur with a close relative of the exotic plant that may share its defensive traits) [11]–[13], or the invasiveness [14] of the non-native plant. If specialist consumers (often insects) are most important, then enemy release may be common following invasion, but if generalist consumers (often vertebrates or larger invertebrates) are most important, then non-native plants may experience biotic resistance [10]. Studies assessing the relative impacts of specialist versus generalist herbivores are uncommon, but the limited contrasts presently available suggest that generalist consumers have greater effects on plant fitness and community composition [9], [15], [16]. However, the relative impacts of specialist (usually insects) and generalist herbivores (vertebrates, non-insect invertebrates, etc.) can shift among studies conducted under different conditions and locations (especially if studies are conducted where larger generalist vertebrates have been removed or excluded). Much of this distinction between effects of generalist versus specialist herbivores depends on how generalists react to new plants. If they commonly fail to recognize novel plants as suitable foods, then they will minimally damage non-native plants; however, if they commonly attack non-native plants and if these plants have not been selected to deter or tolerate these herbivores, then non-native plants may suffer considerable damage and be disadvantaged relative to similar native plants [9], [10], [17]. Recent meta-analysis of field experiments suggests that native herbivores (most impact was by generalists) may selectively feed on exotic plants and that exotic herbivores may selectively feed on native plants [9]; both patterns suggesting that generalist herbivores may preferentially attack naïve plants that have not been selected to deter these herbivores. However, direct evaluations of herbivore preferences for native versus exotic plants have usually been conducted on only a few herbivores or plants, limiting among-species contrasts and making generalizations difficult [10], [17].

Support for enemy release has come from tests with terrestrial plants demonstrating higher insect damage on native vs exotic species [18] and from tests with a snail that preferred native over exotic plants [19]. Conversely, support for the biotic resistance hypothesis comes from several generalist herbivores (crayfish, slugs, grasshoppers) selectively consuming exotic over native plants in laboratory assays [17] and from a meta-analysis of field experiments demonstrating that native herbivores suppress exotic plants [9]. The latter study suggests that invasive plants are following their native herbivores rather than escaping them. Studies focused on effects of insect herbivores and soil microbes over multiple years suggest that the summed effects of enemies may vary among different enemy types and may be context dependent, thus varying among sites or years [20].

We evaluated the competing hypotheses that generalist herbivores would prefer or reject native vs non-native (to the herbivores) plants by determining feeding patterns of aquatic herbivores from North and South America when offered macrophytes from North America, South America, Australia, and Asia. We also conducted analyses of plant traits (chemical, structural, nutritional) thought to influence herbivore feeding by correlating preference for live plants with 1) preference for plants that had been dried, ground to a fine powder, and imbedded in a gel-matrix (thus removing structural but retaining most chemical and nutritional traits), 2) preference for a food treated with plant extracts (thus varying only chemical traits), or 3) plant protein concentrations. By using a suite of herbivores (apple snails) whose distribution is primarily South American, but that has one species native to the southeastern United States, we were also able to conduct an initial assessment of the possibility that phylogenetic history of the herbivore (the history of South American evolution) overrides recent ecological and evolutionary history (one species' occurrence in only North America) and results in it retaining preferences more similar to its South American relatives. Our findings for feeding choices indicate that both North American and South American herbivores prefer plants that are novel, and thus evolutionarily naïve.

## Results

**Figure 1:**
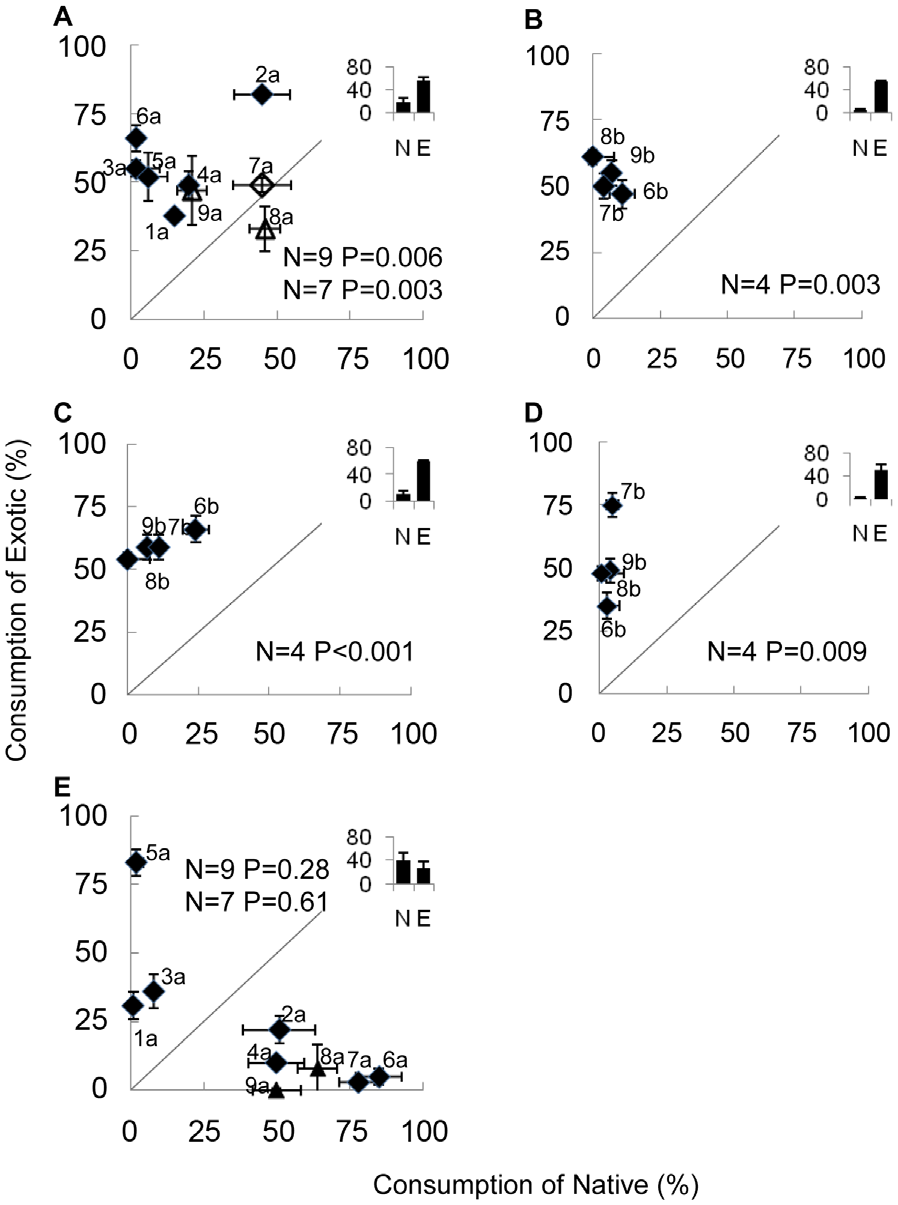
Consumption (mean ±1SE) of confamilial pairs of native vs exotic macrophytes by five herbivore species: (a) *P. spiculifer*, (b) *P. canaliculata*, (c) *P. insularum*, (d) *P. haustrum*, and (e) *P. paludosa*. The sloping line in each figure represents the 50∶50 distribution expected if there is no preference for native versus exotic plants. The filled-in symbols indicate significant preference for one plant in that pair. Inset histograms show the mean consumption across exotic and native plant pairings. P-values from two-tailed paired T-tests are for the pooled histogram data. The triangles present in a) and e) represent comparisons including *Ludwigia grandiflora* and *Pistia stratiotes*, plants whose native distribution is in question. P-values for these two graphs are provided with (N = 9) and without (N = 7) these two data points. The a and b's designate comparisons from the North American and South American perspective, respectively.

**Figure 2:**
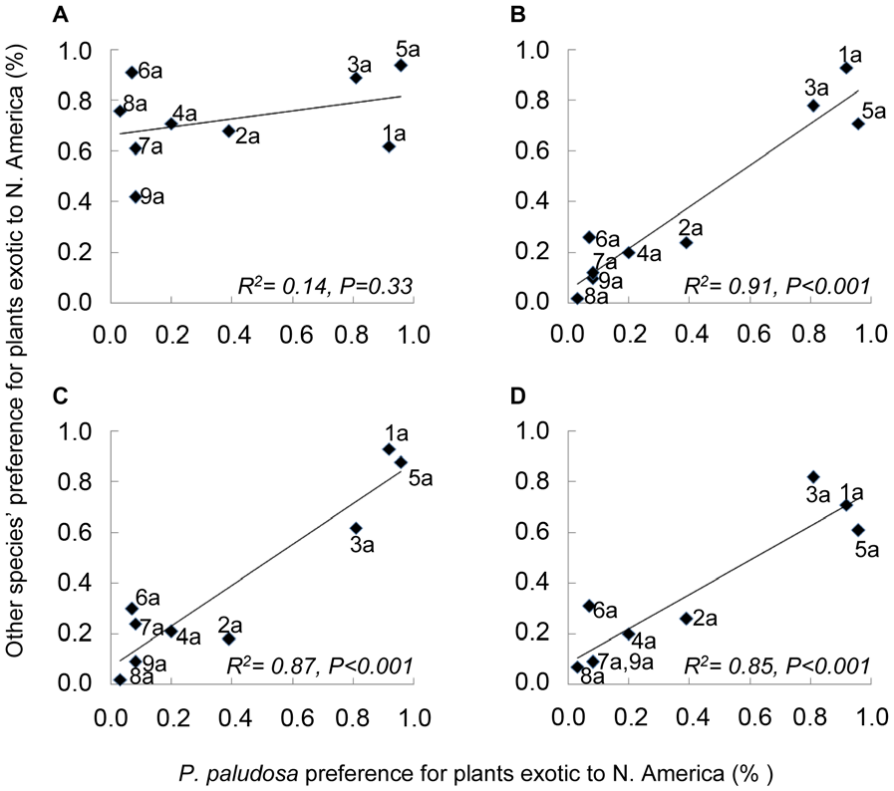
Plant preferences of the native *P. paludosa* correlated with preferences of the other herbivores. Preferences for all species were calculated as the percentage of plant consumed that was exotic to North America. Linear trend-lines and associated R^2^ and p-values are provided. (a) *P. spiculifer*, (b) *P. canaliculata*, (c) *P. insularum*, (d) *P. haustrum.*

To test each herbivore's preference for natives vs exotics across all plant pairings, we first used the mean of each native-exotic plant pair assay as a single replicate and tested the herbivore's response across all plant pairings rather than within each plant pairing alone (the inset histograms in Fig 1 show the pooled means for these contrasts). We also tested for a significant feeding preference within each plant pairing; these are shown as diamond and triangle symbols plotted in Figure 1. If plotted points fall above the diagonal line in Figure 1, then herbivores tend to prefer exotics, below the diagonal indicates a preference for natives, and a scatter along the diagonal indicates no consistent preference. When offered confamilial pairs of native and non-native plants, the crayfish *P. spiculifer* consumed 136% more exotic versus native plant material (df = 8, P = 0.006; Figure 1a); this preference persisted when the two plants with questionable distributions were excluded (feeding on exotics was 195% greater; df = 6, P = 0.003; Figure 1a). In six of the nine plant pairings, there was a significant preference for the exotic plant; there was never a significant preference for the native plant. The three South American snails each demonstrated a 4.5–16.3 fold preference for exotic (to them) North American over native South American plants (df = 3, P = <0.001 to 0.009; Figure 1b-d); all South American snails significantly preferred the exotic in every pairing of native versus exotic plants. The single apple snail native to North America (*P. paludosa*) showed no general preference for native versus exotic plants (df = 8, P = 0.28; or df = 6, P = 0.61, Figure 1e) when plants were considered as native or exotic to the Southeastern United States (where this species occurs). This species exhibited a significant preference within each native-exotic pairing of related plants, but these preferences were sometimes for natives, sometimes for exotics, and thus cancelled each other out in the contrast of the pooled data. When fed all plant pairings, all *Pomacea* snails showed the same significant preference in 92% of the 36 comparisons (9 plant pairs x 4 snails); preferences of the North American snail, *P. paludosa,* were correlated with preferences of the three South American congeners (Figure 2 b-d) but not correlated with preferences of the North American crayfish (Figure 2a).

The crayfish *P. spiculifer* showed no correlation between live plant preference and preference toward ground plants or preference toward extracts from these plants (N = 8, r^2^ = .16, P = 0.33; and N = 8, r^2^ = 0.00, P = .94, respectively) suggesting that this species is responding to plant structural characteristics and not strongly affected by plant chemical traits. Conversely, correlations between live plant preference and preference toward ground plants were significant for both snail species (N = 8, r^2^ = 0.96, P<0.001; N = 8, r^2^ = 0.83, P = 0.002; for *P. paludosa* and *P. insularum*, respectively) while correlations between preference for live plants and preference toward extracts from those plants were significant for *P. insularum* (N = 8, r^2^ = 0.64, P = .02), and nearly so for *P. paludosa* (N = 8, r^2^ = 0.45, P = .07). These patterns suggest that the snails are more strongly affected by plant chemical traits. None of the tested species showed a correlation between preference for intact plants and the protein concentration of the test plants (N = 8, r^2^ = 0.05, P = 0.60; N = 8, r^2^ = 0.05, P = 0.59; N = 8, r^2^ = 0.24, P = 0.21; for *P. paludosa*, *P. insularum* and *P. spiculifer*, respectively).

## Discussion

Both the crayfish native to North America and the three snails native to South America preferred exotic plants over plants from their native ranges (Figure 1). However, the lack of a general preference by the North American apple snail (*P. paludosa)* for either native or non-native species and its preferences correlating closely with those of South American apple snails, suggests that 1) the preferences of *P. paludosa* result more from evolutionary lineage than recent ecology, or 2) that there are general feeding preferences of snails that occur despite differences in native ranges and recent evolutionary history. Thus, plants invading North American from South America will not only be attacked selectively by North American generalist herbivores that they are not evolved to resist, but also (at least in South Florida) by a North American herbivore whose feeding choices mirror those of its South American relatives. We measured feeding preference in the lab rather than demographic impact in the field, but previous studies showing a preference of native generalist herbivores for non-native plants [17] have been consistent with measured impacts of native versus non-native herbivores in the field [9].

Studies on herbivore impact in the field have often focused on insect herbivory, and insects tend to be more specialized in their feeding than do vertebrates or aquatic invertebrate herbivores; insects also commonly have lesser impacts on plant populations and communities than do the more generalist feeders (see [21] for marine, [22] for freshwater, and [10], [15] for terrestrial overviews). Our focus on generalist herbivores from aquatic systems might contrast with patterns generated by more specialized insect herbivores [10], [21]. Additionally, some field studies focusing on herbivore impacts have been conducted in habitats where many native vertebrate herbivores would be excluded due to fencing, hunting, or habitat change associated with urbanization; all biasing for effects of insects (more specialized feeders) and against detecting the natural impacts of larger, generalist herbivores. However, herbivory [via both escape from co-evolved specialist herbivores, and suppression by newly acquired generalist herbivores] is not the only determinant of plant invasions: invasion success also will be affected by competition, disturbance, and the physical traits of the habitat being invaded [10], [18], [20], [23], [24], [25].

Our results support the hypothesis that native, generalist herbivores will constitute biotic resistance to plant invasions. Enemy release would have predicted the opposite trend for these generalist herbivores–that native herbivores would avoid non-native plants due to lack of recognition or because these plants possessed novel traits that native herbivores had not been selected to tolerate [6], [7]. We found no evidence that these herbivores avoided exotic plants due to lack of recognition or due to those plants possessing novel defenses. Other recent studies assessing large sample sizes of native versus non-native plants also have failed to document more effective defenses of invading vs native plants [26]. Our results are for generalist herbivores and for preferences in the lab, not for demographic impact on plants by all herbivores in the field. Under field conditions the relative effects of gaining generalist herbivores could be countered by the advantages of losing co-evolved specialist herbivores (usually insects), but the limited data available to date suggests that generalist herbivores commonly have greater demographic impact on plants [15], [16], [17], [21], [22]

Our results are consistent with other recent studies [6], [9], [17] demonstrating that native, generalist herbivores prefer non-native plants that could not have been selected to deter these consumers. However, some studies have found herbivores preferring native over exotic plants [18], [19], [27] or mixed preferences under different circumstances or by different groups of plant enemies [20]. Carpenter and Cappuccino [18] suggest that studies not supporting the enemy release hypothesis may have included less-invasive species that would have obscured the results. In support of this, Cappuccino & Arnason [28] found that invasive plants were more likely than non-invasive relatives to experience reduced herbivory and to have unique chemical defenses. Our findings are unlikely to be explained by this hypothesis given that many of the exotic species we utilized are highly invasive. On average, the non-native plants we used are listed as a weed for 6±6 U.S. states (Ranges from 0 for *M. simulans* to 21 for *Hydrilla*), and one plant (*Eichhornia*) is listed as one of the 100 worst invasive species [29]. Additionally, a meta-analysis of field experimental results failed to find a relationship between plant invasiveness and herbivore impact [9] and a recent contrast across numerous native and exotic plants failed to find consistent differences in the deterrent properties of native versus exotic plants [26].

In addition, investigators documenting support for the enemy release hypothesis note that preference for natives accounts for a very small percentage of the variance in results [18] and may not lead to differential mortality [30]. This suggests that while low palatability of exotics may be important in some cases, it is not a primary mechanism accounting for the spread of invasive plants [26]. Other characteristics besides, or in conjunction with, palatability have been found to be important for the establishment and spread of exotics including tolerance to grazing [31], faster growth or higher fecundity [32], [33], a positive response to disturbance [25], and invasion melt-downs where non-native herbivores selectively suppress native plants and facilitate invasion by non-native plants that have evolved with these invasive herbivores [9].

We note that our study tested confamilial pairs of native and exotic plants. Research suggests that herbivore familiarity with a relative of the invasive species can impact preference because relatives may have similar chemical and structural defenses. However, there is conflicting information on the direction of this relationship. Some studies indicate that herbivores avoid phylogenetically novel plants [12], [13] while others indicate they prefer such plants [11], [34]. Both Hill and Kotanen and Dawson et al. [12], [13] found higher herbivore damage on exotic plants that had close relatives within the invaded range. Conversely, Hokkanen and Pimentel [34] found that successful biological control agents were often novel enemies who have no history of co-evolution with the prey they control. Additionally Ricciardi and Ward [11] show that exotic plants without native congeners have a lower survival when compared to exotic plants with native congeners. This discrepancy in results could be due to differing methodology: both Hill and Kotanen and Dawson et al measured leaf damage by insects, but Hokkanen and Pimental and Ricciardi and Ward examined plant survival [11], [12], [13], [34]. When herbivores affect plant survival by removing entire plants, this does not leave a record of their effect (leaf damage) and may result in a biased estimate of impact when leaf damage alone is assessed.

There was no correlation in plant preference between the one snail native to North America (*P. paludosa*) and the North American crayfish *P. spiculifer*; however, there were significant correlations between the preference of *P. paludosa* and the three South American snails. The strongest correlations were between the North American snail (*P. paludosa*) and its closest relatives in South America-*P. insularum* and *P. canaliculata* [35], suggesting that feeding choices of *P. paludosa* may have been affected by evolutionary inertia. No estimate exists as to when *P. paludosa* split from the rest of the Pomacea family, but the close genetic relationship between *P. paludosa* and *P. insularum and P. canaliculata* [35], [36] suggests a recent divergence. These results agree with earlier assertions that phylogenetic history can impact herbivore preferences [12]–[14]. However, previous studies have concentrated on the phylogenetic history of the exotic prey; we note this reasoning also extends to the phylogenetic history of the native consumer.

Our results show that both generalist crayfish and snails preferred exotic over native plants even though they responded to different plant traits, with crayfish most affected by plant structural traits (i.e., preference patterns for live plants changing once the plants are dried and ground [37]) and snails responding more to plant chemical traits (i.e., the consistent preferences across live plants, ground plants, and plant extracts). Neither crayfish nor snails showed a correlation between plant preference and protein content, suggesting that protein (which commonly limits some herbivores [38]) had minimal influence on these feeding choices. It would be interesting to test whether preferences of South American crayfish align with the preferences of the South American snails or the North American crayfish to see if phylogeny or geography more strongly influences preference in response to structural or chemical traits, respectively.

In summary, we document patterns supporting the hypothesis that native generalist herbivores will produce biotic resistance to plant invasions. Both North American crayfish and South American snails preferred exotic plants over confamilial natives, despite responding to different plant characteristics. The single species of apple snail that occurs in North American showed no preference for native or exotic plants from a North American perspective, but instead exhibited preferences that correlated with its history of evolution in South America. This suggests that phylogenetic legacy will affect choices of the herbivore as well as resistance or susceptibility of host plants.

## Materials and Methods

### Collections

Crayfish and apple snails are omnivores that can strongly impact freshwater habitats [39], [40]. The crayfish, *Procambarus spiculifer*, is native to the southeastern United States (including Mississippi, Alabama, Florida, Georgia, and South Carolina) [41]. Adult crayfish were collected from the Chattahoochee River in Atlanta, Georgia, USA. The offspring from these crayfish were fed commercial herbivore food and frozen shrimp until large enough for utilization in bioassays. All apple snail species are currently present in South Florida, but three are native to South America: *Pomacea canaliculata* to Argentina, Bolivia, Paraguay, Uruguay and Brazil; *Pomacea haustrum* to Brazil, Peru and Bolivia; and *Pomacea insularum* to Argentina, Brazil, Bolivia, Uruguay and Paraguay), and only one species (*Pomacea paludosa*) is native to North America [36]. *Pomacea paludosa* and *P. insularum* were collected as eggs; *P. insularum* from Lake Lure, Georgia (N 31° 33.210′ W 82° 28.947′) and Lake Tohopekaliga, Florida (N 28° 13.033 W 81° 22.533), and *P. paludosa* from Lake Tohopekaliga, Florida. Adult *P. canaliculata* were obtained from Neighborhood Fish Farm in Miami, Florida, and adult *P. haustrum* were obtained from Paradise Aquatics in Winterhaven, Florida. Both of these species produced viable eggs that hatched in the lab. All snails used in experiments were hatched between 2 June and 29 July 2008. Because adult and juvenile snail species are difficult to identify, all species were identified according to characteristics of their eggs [36], and juveniles were held separately in labeled tanks. Snails were reared on lettuce until they reached a size where they could be utilized in assay experiments. Crayfish were housed individually in 946 ml containers placed in a 180×90 cm flow-through water table. Snails were housed in 38 L tanks until used in feeding assay; for assays, they were transferred to 946 ml containers. Replicates of all assays were in separate containers to assure independence.

Nine pairs of confamilial native and exotic plants were utilized (Table 1). Distributions (native vs exotic) were determined using the USDA Germplasm Resources Information Network (GRIN) [42] as this was the best reference for North and South American plants. There is uncertainty surrounding the native distribution of *Ludwigia grandiflora* and *Pistia stratiotes*. Both species are listed as non-native by the Atlas of Florida Vascular Plants [43], and were considered exotic by Parker and Hay [17]; however, GRIN lists them as native to S. Florida. Results are thus presented both with and without these comparisons. Plants were considered native to the South American snails if the native distribution of the snail overlapped with the native distribution of the plant. Two of the plants considered “exotic” to the South American snails were listed as native in either Colombia or Venezuela. As *Pomacea* are not listed as native in these countries, we assumed there was no historical overlap of *Pomacea* apple snails with these plant species and that they would be “novel” to the snails. We were able to collect nine pairs of related plants where one was native to North America and one was exotic (see Table 1). Only four of these nine pairs represented a native and an exotic species pairing from the perspective of the South American herbivores (Table 1, see those with a “b” designation). When possible, related pairs of plants were collected from the same location to minimize confounding effects due to local conditions (see Table 1), however, this was not possible for four of the comparisons. All plants were either used within 24 h of collection or planted in 72 L tubs and grown in a greenhouse at the Georgia Institute of Technology until needed.

**Table 1:** Confamilial plant pairs used in feeding assays with information on native distributions [36].

COMPARISON	NATIVE PLANT	NATIVE DISTRIBUTION	EXOTIC PLANT	EXOTIC DISTRIBUTION
1a	Pontederia cordata^1^	US,Brazil, Bolivia, Argentina, Paraguay, Uruguay, Colombia, Equador	Eichhornia crassipes	Venezuela, Brazil, Guyana, Suriname
2a	Myriophyllum pinnatum^2^	US, Canada, Africa, Asia, Europe	Myriophyllum simulans^2^	Australia
3a	Orontium aquaticum^2^	US	Colocasia esculenta^4^	Tropical Asia
4a	Peltandra virginica^3^	Canada, US	Colocasia esculenta^4^	Tropical Asia
5a	Vallisneria americana^5^	US, Meso America, Venezuela	Hydrilla verticillata^5^	Asia
6a&b	Vallisneria americana^5^	US, Meso America, Venezuela	Egeria densa^5^	Brazil, Argentina, Uruguay
7a&b	Myriophyllum heterophyllum^2^	US	Myriophyllum aquaticum^2^	Brazil, Argentina, Bolivia, Equador, Peru, Chile, Paraguay
8a&b	Peltandra virginica^3^	Canada, US	Pistia stratiotes^2^	FL, TX, Africa, Brazil, Argentina
9a&b	Ludwigia palustris^3^	US, mexico, Costa Rica, Guatemala, Colombia	Ludwigia hexapetala^6^	FL, SC. TX, Guatemala, Brazil, Paraguay, Argentina

1 collected at Clayton County Water Authority.

2 ordered from Arizona Aquatic Gardens.

3 collected in the Chattahochee River.

4 sent from Texas.

5 collected from Lake Lanier.

6 collected from Piedmont College.

“a” and “b”denote comparisons from the North American and South American perspectives, respectively.

### Assays

Pieces of confamilial native and exotic plants were matched by surface area and mass and offered to herbivores in 946 ml containers. Assays were grouped into 10 blocks of replicates, where each block included one replicate of each herbivore species plus one control to monitor autogenic changes in plant mass unrelated to feeding [44], [45]. Because data were analyzed by species, blocks were used to correct treatment plants for autogenic changes of control plants within that block, but a block factor could not be included in the analysis. Plant starting masses were corrected for autogenic change according to the formula: T_i_ x (C_f_/C_i_), where T_i_ is the initial mass of plants available for consumption by the herbivores and C_i_ and C_f_ are the initial and final masses of the plants from the matching controls [45]. All pieces within each block were cut from the same plant when possible, and no individual plant was used in more than one block. After 50% of one of the plant species was consumed or after 5 days (whichever happened first) the assay was stopped for that replicate. Remaining plants were blotted and a wet mass determined at the end of the assay. This produced assay durations of 1–5 days for each replicate depending on the rate of feeding. If no consumption occurred by 5 days or if all of both plants were consumed between monitoring periods, that replicate was discarded because it provided no information on relative preference. Paired T-tests evaluated differences in consumption for each native vs exotic contrast. A second paired t-test using the mean from each paired contrast as a single replicate, evaluated the overall preference of each consumer for native versus exotic plants.

We were also interested in determining if plant palatability was correlated with plant structural, chemical, or nutritional traits. Due to a limited amount of plant matter, we were unable to run these tests with all herbivore species, so included the crayfish species (*P. spiculifer*), the North American snail species (*P. paludosa*) and the fastest feeding South American snail species (*P. insularum*). To destroy structural traits but retain chemical and nutritional traits, plants were freeze-dried, ground with a Wiley Mill until particles could pass through a 60um mesh, and these ground particles reconstituted into a gel-based food [46]. To assess the effects of chemical traits unrelated to structural and nutritional traits, freeze dried plants were extracted 3–4 times for 1–2 h each time in a 2∶1 mixture of dichloromethane: methanol and this extract coated onto freeze-dried and finely ground lettuce to create an artificial food in a gel-matrix [47]. Masses of lettuce and extract were varied so as to match the dry mass per volume of the natural plants being evaluated. Plant densities (dry mass/volume) were calculated by measuring volumetric displacement of live plant tissue and mass of the associated freeze dried material to calculate g/ml (N = 5 per plant species). The agar gel recipe included mixing 3 ml of deionized water, enough ground plant matter to equal 10 ml of live plant, and then 0.19 g of agar in 7 ml of boiling deionized water [48]. Agar and plant mixtures were combined and quickly spread into either a fiberglass mold with window screen underneath [46] or into assay “dominoes.” Dominoes were 102 by 55 mm pieces of flat PVC with 30 3 mm wide by 1 mm deep indentations drilled into opposite halves of each block. The warm agar food was scraped into the indentations where it hardened as it cooled. The native and exotic plants being compared were randomly assigned to opposite ends of a domino and ends labeled to allow identification at the end of each bioassay. Feeding was quantified as the number of indentations from which crayfish removed and consumed the food. Dominos proved to be a good methodology for crayfish, whose sloppy feeding sometimes makes measurement of consumption from fiberglass screen gels difficult. The fiberglass mold was appropriate for apple snails because their radulas could more effectively graze from the flat surface of the gel than from the holes in the dominoes and the grid of the screen made it easy to assess feeding as the number of grid squares from which snails had consumed the artificial food.

Preferences were converted to a single number by calculating the proportion consumed that was exotic (grams of exotic consumed divided by the sum of the grams of exotic and native plant combined). Correlations were completed between the results from the live plants and ground plants or live plants and extracts from the plants to determine the influence of structural and chemical characteristics. Similarly, protein content was measured using a modified Bradford assay [49] and correlated with live plant preferences. This provides a crude measurement of the importance of structural, nutritional (as measured by protein) and chemical characteristics.
